# Intra‐fractional patient motion when using the Qfix Encompass immobilization system during HyperArc treatment of patients with brain metastases

**DOI:** 10.1002/acm2.13143

**Published:** 2021-03-03

**Authors:** Riho Komiyama, Shingo Ohira, Hikari Ueda, Naoyuki Kanayama, Akira Masaoka, Masaru Isono, Yoshihiro Ueda, Masayoshi Miyazaki, Teruki Teshima

**Affiliations:** ^1^ Department of Radiation Oncology Osaka International Cancer Institute Osaka Japan; ^2^ Department of Medical Physics and Engineering Osaka University Graduate School of Medicine Suita Japan

**Keywords:** HyperArc, intra‐fractional patient motion, QFix EncompassTM immobilization system, stereotactic radiosurgery, stereotactic radiotherapy

## Abstract

**Purpose:**

This study investigated the intra‐fractional motion (IM) of patients immobilized using the QFix Encompass Immobilization System during HyperArc (HA) treatment.

**Method:**

HA treatment was performed on 89 patients immobilized using the Encompass. The IM during treatment (including megavoltage (MV) registration) was analyzed for six degrees of freedom including three axes of translation (anterior‐posterior, superior‐inferior (SI) and left‐right (LR)) and three axes of rotation (pitch, roll, and yaw). Then, the no corrected IM (IM_NC_) was retrospectively simulated (excluding MV registration) in three directions (SI, LR, and yaw). Finally, the correlation between the treatment time and the IM of the 3D vector was assessed.

**Results:**

The average IM in terms of the absolute displacement were 0.3 mm (SI), 0.3 mm (LR) and 0.2° (yaw) for Stereotactic radiosurgery (SRS), and 0.3 mm (SI), 0.2 mm (LR), and 0.2° (yaw) for stereotactic radiotherapy (SRT). The absolute maximum values of IM were <1 mm along the SI and LR axes and <1° along the yaw axis. The absolute maximum displacements for IM_NC_ were >1 mm along the SI and LR axes and >1° along the yaw axis. In the correlation between the treatment time and the IM, the *r*‐values were −0.025 and 0.027 for SRS and SRT respectively, along the axes of translation. For the axes of rotation, the *r*‐values were 0.012 and 0.206 for SRS and SRT, respectively.

**Conclusion:**

Encompass provided patient immobilization with adequate accuracy during HA treatment. The absolute maximum displacement IM was less than IM_NC_ along the translational/rotational axes, and no statistically significant relationship between the treatment time and the IM was observed.

## 1 INTRODUCTION

Brain metastases are a common cause of morbidity and mortality in patients suffering from a variety of solid tumors and they affect 20–40% of cancer patients. As primary cancer management has improved, survival times have increased but so has the incidence of patients developing brain metastases.^1^, ^2^ Stereotactic radiosurgery (SRS) and stereotactic radiotherapy (SRT) deliver high doses of radiation to tumors in cranial lesions as a single fraction SRS or multiple fractions SRT and are gaining popularity for the treatment of brain metastases. SRS is now widely used for the treatment of patients with four or less brain metastases and a life expectancy >3–6 months.^3^, ^4^ However, Hughes et al. showed that the overall survival rate for patients with 5–15 brain metastases was similar to that of patients with 2–4 brain metastases when they received SRS.^5^ Multi‐isocenter irradiation techniques for multiple targets, such as Gamma Knife which is performed using a rigid and invasive fixed head ring, ^6^ require a treatment time approximately proportional to the number of treated lesions. In practice, the treatment time ranges from 20 min for a single lesion to >1 h for multiple lesions.^7^

HyperArc™ (HA) (Varian Medical System, Palo Alto, CA, USA) provides single isocentric irradiation using a non‐coplanar volumetric modulated arc therapy (VMAT) technique. This is combined with a simple treatment planning procedure that includes automated settings for the collimator angles, non‐coplanar beam arrangement, and isocenter location. HA plans improve tumor conformity and reduce the dose of radiation applied to surrounding tissue.^8^ Moreover HA requires a shorter treatment time than the conventional VMAT technique because it incorporates automated delivery (e.g., couch automation). Furthermore, for the treatment to be automated, the QFix Encompass^TM^ immobilization system must be included in the HA treatment plan. The Encompass is a frameless‐mask based system with a clam‐shell style mask that was created by QFix (Avondale, PA, USA).^9^ It allows the patient to be placed in an optimal position that ensures machine clearance during automated delivery.

It is necessary to set a small tumor margin for SRS and SRT treatment planning because the risk of radionecrosis increases with the gross tumor volume and despite local controls with a large margin there is no significant difference compared to a small margin.^10^ A patient’s position is decided in six axes using corn‐beam computed tomography (CBCT) for image guided radiation therapy. However, SRS and SRT requires a relatively long treatment time (approximately 20 min) and intra‐fractional patient motion (IM) may occur during dose delivery. IM may result in underdosing of the target or overdosing of the surrounding normal tissue.^11^ Minniti et al. showed that IMs of up to 3 mm occurred when using frameless stereotactic systems.^6^ However, the IMs when the Encompass is used during HA dose delivery have not been reported.

Therefore, this study aims to investigate IM during HA treatment when the patients are immobilized using the Encompass. Furthermore, images before, during, and after treatment will be analyzed to assess the necessity of monitoring patient motion.

## 2 MATERIALS AND METHODS

### 2.A Patients and computed tomography (CT) simulation

**FIG. 1:**
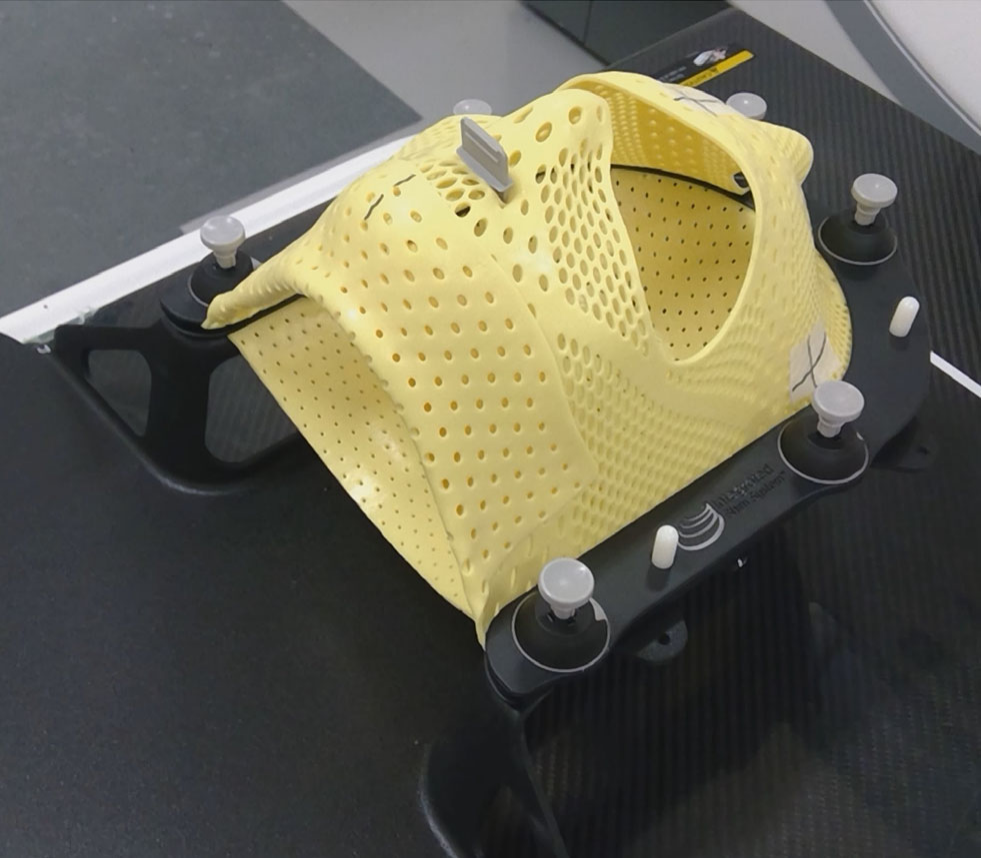
Clam‐shell‐style thermoplastic mask for Encompass, created for HyperArc treatment.

The retrospective study was approved by our ethics committee (No. 18277‐2). It included 89 patients who underwent HA treatment for brain metastases at our institution; 56 of the patients were treated with SRS and 33 were treated with SRT. Of these patients, 40 had one metastasis, 29 had 2–4 metastases, and 20 had 5–19 metastases. The median age at the time of treatment was 65 yr with a range of 30–88 yr. The patients were immobilized using the Encompass and then underwent a CT simulation (Revolution HD; GE Medical Systems, Milwaukee, WI). The Encompass was made from 3‐mm thick perforated thermoplastic and consisted of a posterior section, an anterior open view mask, and a bite block; it locked at six fixation points. (Fig. 1) CT scanning was performed tube voltages of 80 and 140 kVp, tube current of 600 mA, and a volume CT dose index (CTDI_vol_) of 105.54 mGy. The acquisition parameters were a pixel count of 512 × 512, a field of view of 350 mm, and a slice thickness of 1 mm which produced high‐quality reconstructed slices.

### 2.B Treatment planning

The CT images were loaded into a treatment planning system (Eclipse, version 15.6, Varian Medical Systems, PaloAlto, CA). HA plans were generated based on the linear accelerator of a TrueBeam STx and Edge, which provided a multi leaf collimator with a central high resolution leaf width of 2.5 mm (central 8 cm, leaf width projected at isocenter) and an outboard leaf width of 5 mm (outer 14 cm, leaf width projected at isocenter). A clinical target volume was generated by adding an isotropic margin of 0–2 mm to the gross tumor volume and the planning target volume was generated by adding an additional 1 mm margin. The prescription dose was 20–24 Gy in a single fraction and 7–10 Gy in 3–5 fractions for 95% of the planning target volume for SRS and SRT, respectively. Each plan was designed for a 6‐MV photon beam or flattening filter free beams with a 6‐MV photon beam energy at maximum dose rates of 600 and 1400 monitor units per minute (MU/min). Overall, 3–4 arc fields were arranged (one coplanar arc with a 0° couch and noncoplanar arc fields at 315°, 45°, and 90° or 270° couch).

### 2.C Treatment

**FIG. 2:**
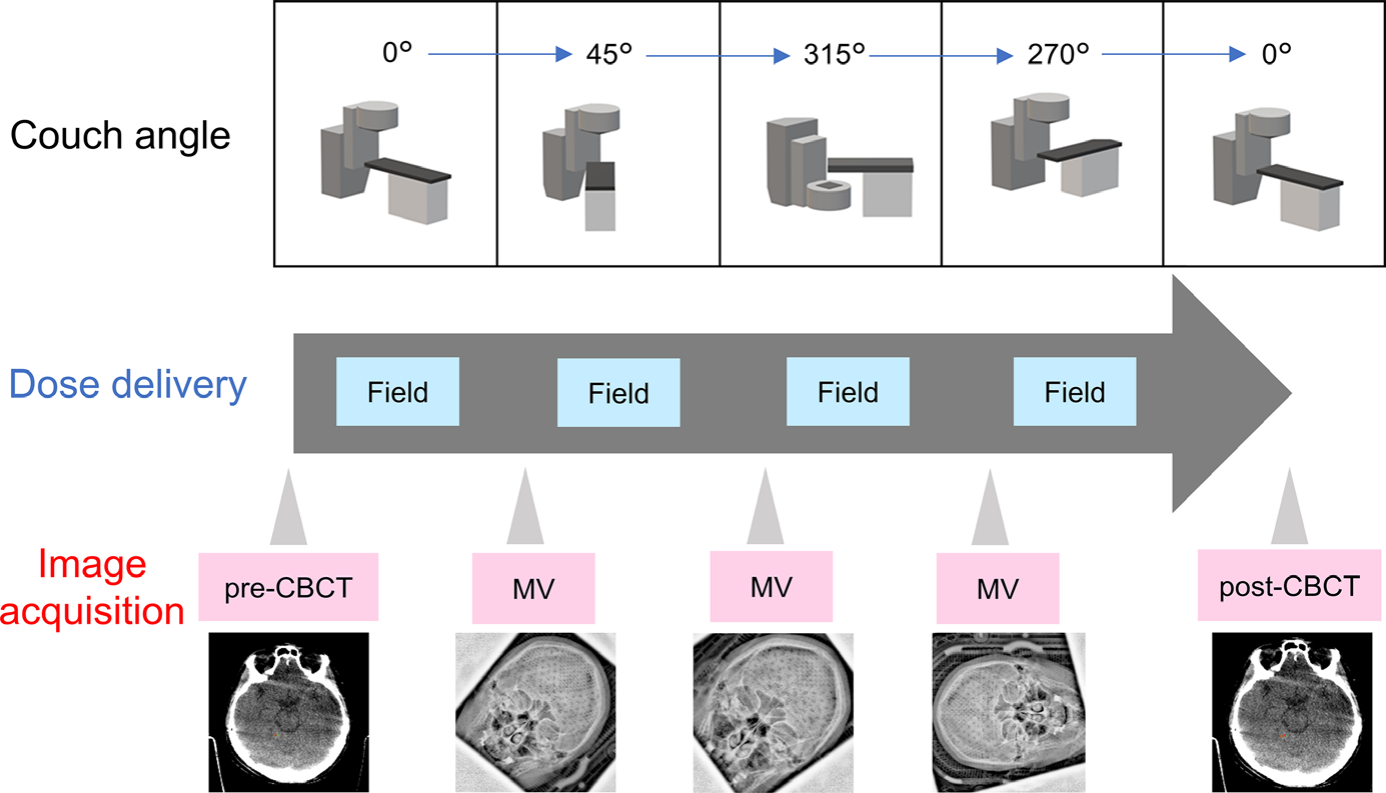
Flowchart of the HyperArc treatment process in this study. corn‐beam computed tomography (CBCT) images were acquired before (pre‐CBCT) and after (post‐CBCT) dose delivery. A megavoltage (MV) image in the anterior‐posterior or posterior‐anterior direction was obtained during treatment and registered with the corresponding DRR image that was generated from the planning CT.

Patients were immobilized using the Encompass as they were in the CT simulation. They were aligned to the isocenter location determined during the HA plan in accordance with the wall‐mounted lasers with mask marks. Before dose delivery, a CBCT image (pre‐CBCT) was acquired using a tube voltage of 100 kVp, tube current of 1200 mA, and CTDI_vol_ of 25.32 mGy with a linear accelerator‐mounted on‐bord imager. The parameters for image reconstruction were a slice thickness of 1 mm, pixel matrix of 512 × 512 pixels, and field of view of 32 cm. The pre‐CBCT images were registered with the CT images used for the treatment plans in the 3D/3D bony matching with six degrees of freedom (three axes of translation: anterior‐posterior (AP), superior‐inferior (SI), and left‐right (LR); three axes of rotation: pitch, roll, and yaw). The images were assessed by radiation oncologists. After the CBCT registration, the doses were delivered with HA, as shown in Fig. 2. A megavoltage (MV) image in the AP or PA direction was acquired before each field using an electronic portal imaging device and the MV image was registered with the corresponding digitally reconstructed radiography (DRR) generated from the planning CT. Finally, CBCT images were acquired after the treatment (post‐CBCT) to assess the IM.

### 2.D Data analysis

The IM during the HA treatment was defined as the difference between the patient’s position before and after CBCT (including MV registration) in six dimensions (AP, SI, LR, pitch, roll and yaw). Subsequently, the no corrected IM (IM_NC_) was retrospectively simulated (excluding MV registration) in three dimensions (SI, LR, and yaw) The 3D IM was calculated as the square‐root of the sum of squares of three translational/rotational IMs. The treatment time was defined as the time between the pre‐ and post‐CBCT image acquisitions. The correlation between the treatment time and the 3D IM was assessed.

We used SPSS (version 24; IBM, USA) for the statistical analyses in this study. Initially, the Shapiro‐Wilk test was performed to measure the normality of the distribution across all the IM and IM_NC_ axes. In cases where the *P < *0.05, measurement were non‐normally distributed. In statistical comparisons between IM and IM_NC_, paired Wilcoxon signed‐rank (non‐normal distribution) and Welch (normal distribution) tests were used for the SI, LR, and yaw axes. In cases where the *P* < 0.05, we rejected the null hypothesis that there was no difference between IM and IM_NC_. In addition, Levene’s test was performed to statistically assess the equality of variance between IM and IM_NC_ along the SI, LR, and yaw axes. In cases where the *P < *0.05, the variance was regarded as unequal. Furthermore, the Pearson correlation coefficient *r* was used to determine if there was any linear correlation between the variables (treatment time and 3D IM). An *r* < 0.4 indicates a weak linear relationship, and an *r* > 0.4 indicates a strong linear relationship. A *P < *0.05 was considered to indicated statistical significance.

## 3 RESULTS

**FIG. 3:**
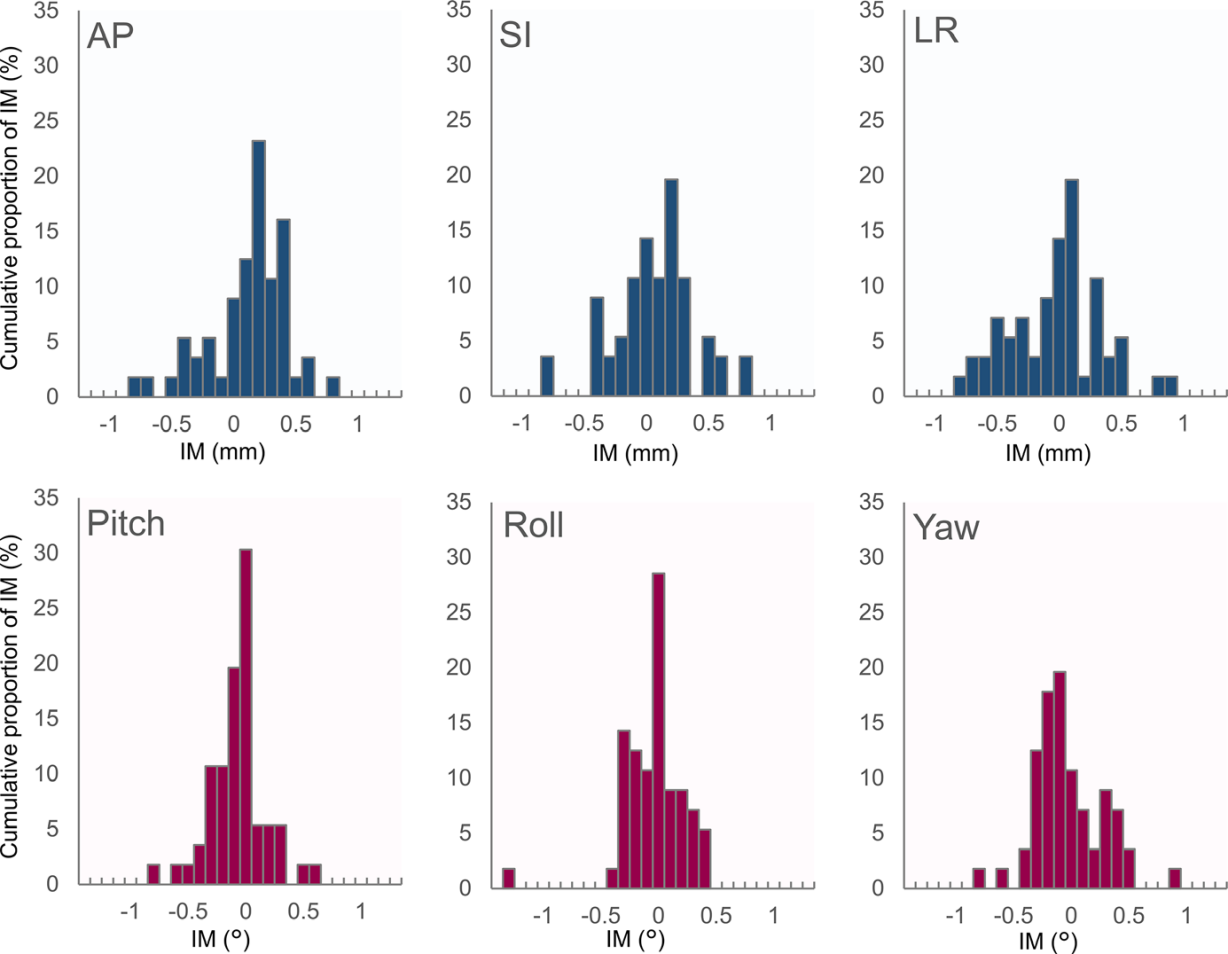
Histograms of the intra‐fractional motion along the translational/rotational axes for Stereotactic radiosurgery.

**FIG. 4:**
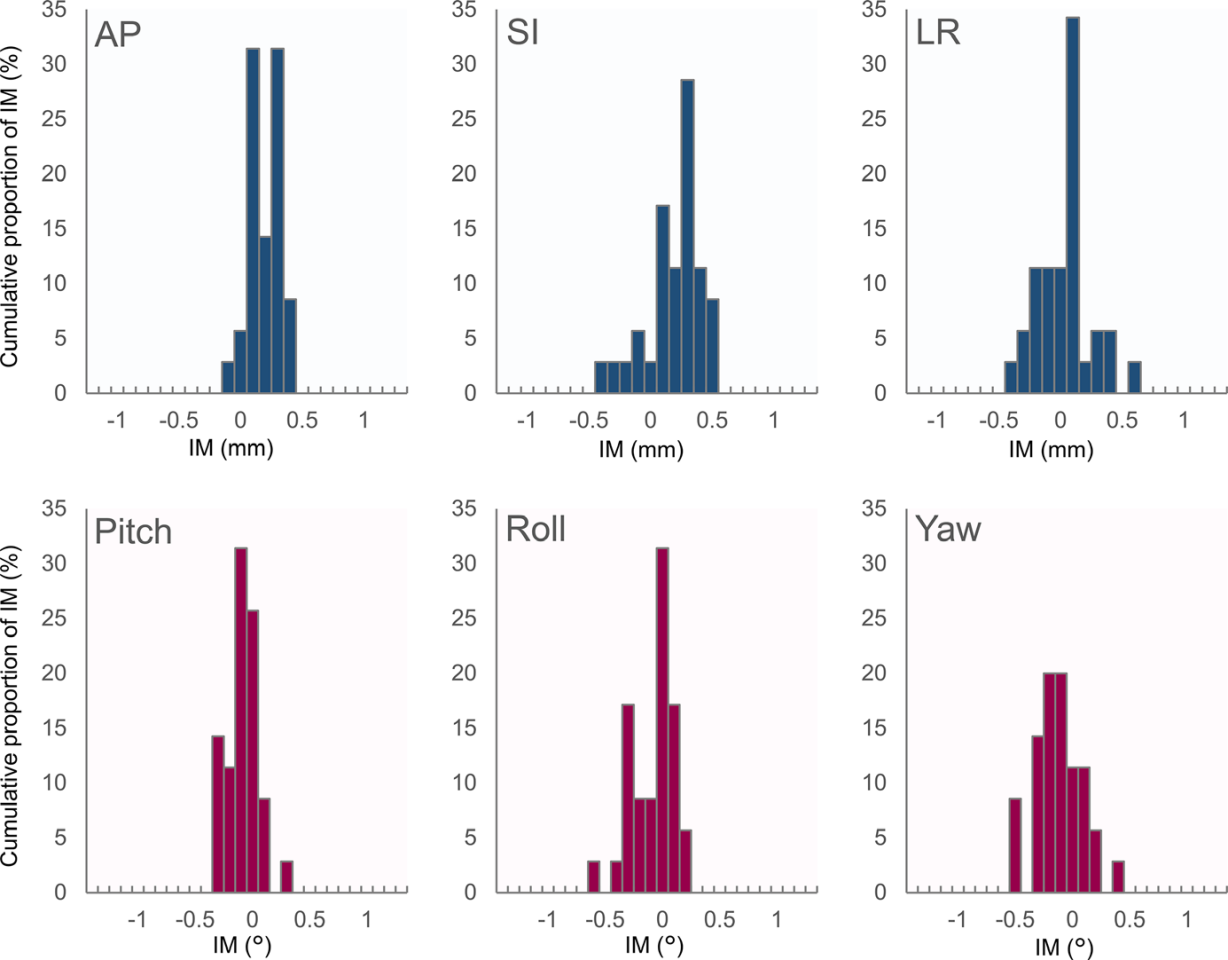
Histograms of the intra‐fractional motion along the translational/rotational axes for stereotactic radiotherapy.

Figures 3 and 4 show histograms of the IM along each axis for SRS and SRT, respectively. IMs were normally distributed along SI, LR, pitch and yaw axes (*P* > 0.05), but not along AP and roll axes (*P* < 0.05) for SRS, and IMs were normally distributed along all axes for SRT (*P* > 0.05). The normality of the distribution was confirmed along all IM_NC_ axes for SRS, but not for SRT. The average and standard deviation (SD) of numerical results are reported in the following. Average values of IM and IM_NC_ in terms of the absolute displacement along all axes are shown in Table 1. The SD of IM_NC_ was larger than that of IM for both SRS and SRT along the SI, LR, and yaw axes. There was a significant difference between IM and IM_NC_ in SRS (*P* < 0.01) and SRT (*P* < 0.01) along the SI axes. There was no significant difference in the average IM along the LR and yaw axis in SRS (*P* > 0.05) and SRT (*P* > 0.05). The absolute maximum values of IM were <1 mm along the SI and LR axes and <1° along the yaw axis. However, the absolute maximum displacements for IM_NC_ along the SI and LR axes were 1.5 and 1.7 mm in SRS, and 1.6 and 1.2 mm in SRT, respectively. Along the yaw axis, the absolute maximum shifts in IM_NC_ were >1° in several cases of IM_NC_ in both SRS and SRT. Furthermore, in the AP direction, the absolute maximum IM displacement was 0.8 and 0.4 mm in SRS and SRT, respectively. In most of the cases, the absolute maximum shifts in IM were <0.5° along the pitch and roll axes for SRS and SRT, as shown in Figs. 3 and 4, respectively.

**TABLE 1:** Average values of intra‐fractional motion (IM) and IM_NC_ in terms of the absolute displacement along all axes.

	SI	LR	Yaw
	Average± SD (maximum) mm		Average± SD (maximum)°
SRS			
IM	0.3 ± 0.2 (0.9)	0.3 ± 0.2 (0.9)	0.2 ± 0.9 (0.9)
IM_NC_	0.4 ± 0.3 (1.5)	0.4 ± 0.4 (1.7)	0.3 ± 1.3 (1.3)
*T* – *test* *P‐value*	0.002	0.718	0.090
*Levene's test* *P‐value*	0.016	0.012	0.007
SRT			
IM	0.3 ± 0.2 (0.5)	0.2 ± 0.1 (0.6)	0.2 ± 0.2 (0.6)
IM_NC_	0.3 ± 0.3 (1.6)	0.3 ± 0.3 (1.2)	0.2 ± 0.2 (0.8)
*T* ‐ *test* *P‐value*	0.000	0.379	0.109
*Levene's test* *P‐value*	0.000	0.000	0.180

**FIG. 5:**
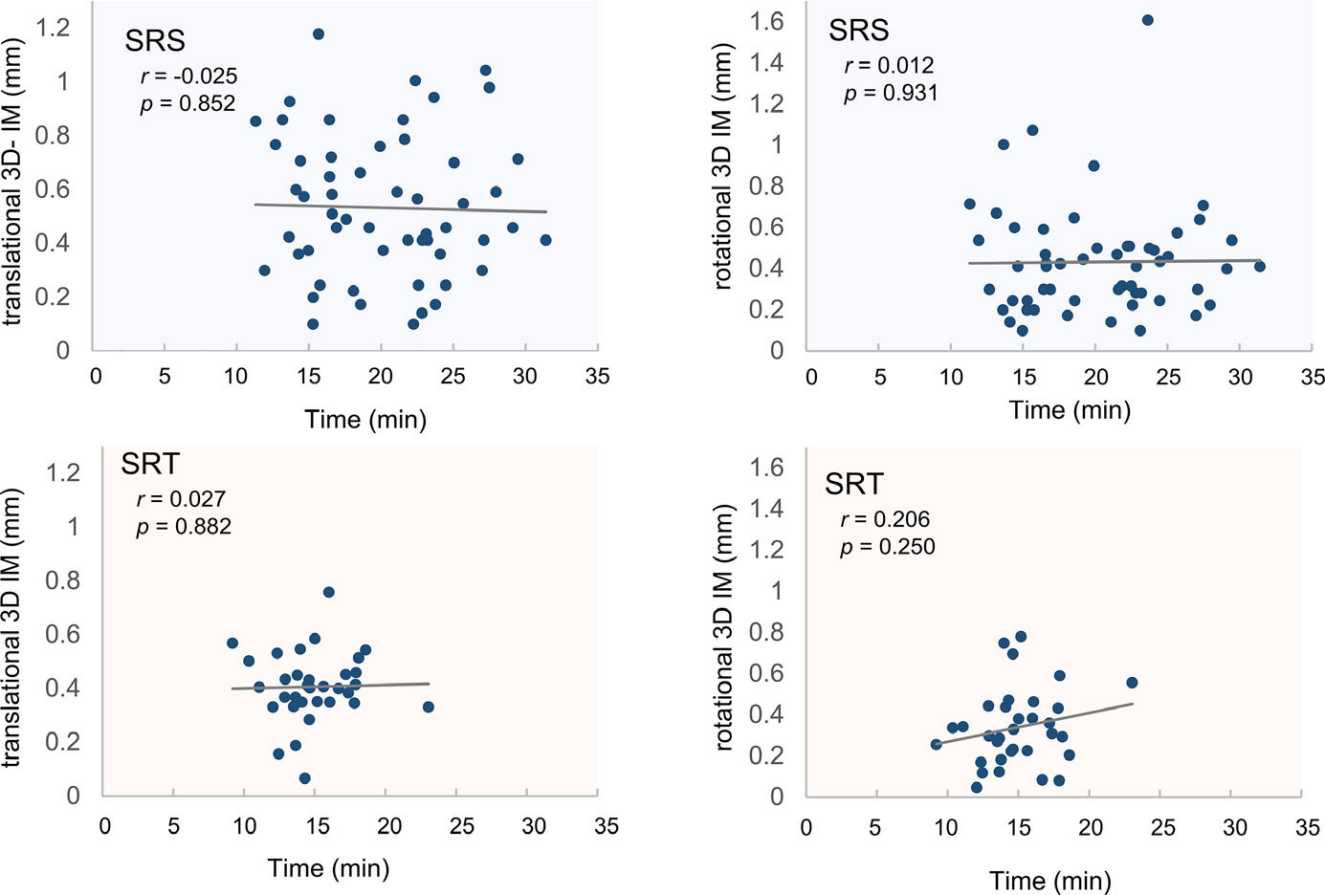
Correlation between the three‐dimensional intra‐fractional motion and the treatment time during Stereotactic radiosurgery and stereotactic radiotherapy.

Average values of treatment time was 20 ± 5 min and 15 ± 3 min for SRS and SRT, respectively. Figure 5 shows the correlation between the 3D IM and the treatment time in SRS and SRT. There was no correlation between the points along the axes of translation, and the *r*‐values were −0.025 (*P* = 0.882) and 0.027 (*P* = 0.882) for SRS and SRT, respectively. For the axes of rotation, the *r*‐values were 0.012 (no correlation, *P* = 0.931) in SRS and 0.206 (weak correlation, *P* = 0.250) in SRT.

## 4 DISCUSSION

Historically, various types of masks have been used to effectively immobilize patients during SRS and SRT. Frame‐mask based systems include rigid and invasive stereotactic head‐rings and they are mainly limited to use with single‐fraction treatments due to their invasive nature.^12^ Consequently, frameless‐mask based systems have grown in popularity since they are noninvasive, provide greater comfort for patients, and allow treatments to be fractionated while maintaining a high standard of immobilization. The Encompass is frameless‐mask based system and an integral part of the HA high‐definition radiotherapy automated SRS delivery workflow.^9^ In this study, for absolute displacements using the Encompass during SRS, the average ± SD of IM for the translation axes were 0.3 ± 0.2 mm (AP), 0.3 ± 0.2 mm (SI) and 0.3 ± 0.2 mm (LR). Giuseppe et al. showed that, for absolute displacements using non‐invasive relocatable frameless‐mask based systems in SRS, the average ± SD of IM were approximately 0.1 ± 0.2 mm (AP), 0.1 ± 0.2 mm (SI) and 0 ± 0.1 mm (LR).^6^ The IM were small in both their study and ours. Furthermore, Ramakrishna et al. demonstrated that a frameless‐mask based system may provide better immobilization than invasive frame based mask systems when an orthogonal x‐ray image‐guidance system is used to correct for IM during treatment.^13^ In our study, the MV image acquisition significantly reduces setup errors because the standard deviation of IM_NC_ is larger than that of IM. Moreover the absolute maximum shifts of IM along the SI and LR axes were <1 mm in both SRS and SRT, but the absolute maximum displacement of IM_NC_ along the SI and LR axes were 1.5 and 1.7 mm in SRS, and 1.6 and 1.2 mm in SRT. Therefore, it is necessary to correct for IM during HA treatment to compensate for doses with a small margin.

In this study, the average IM in terms of the absolute displacements along the rotational axes for SRS were: 0.2 ± 0.2° (pitch), 0.2 ± 0.2° (roll), and 0.2 ± 0.9° (yaw). Tejinder et al. showed that when thermoplastic frameless‐mask based systems were used in SRS, average values of IMs were −0.2 ± 0.5° (pitch), −1.0 ± 1.0° (roll), and −0.3 ± 0.3° (yaw). Therefore, the IM along the roll axis can be reduced by using the Encompass. Furthermore, Roper et al. simulated the dosimetric effects in SRS and their results showed that rotational setup errors may cause non‐negligible underdosage. The rotational errors along the pitch and roll axes, which were >1°, may result in D_95_ (the percentage isodose line relative to the prescription dose that covers 95% of the planning target volume) values <95%.^14^ In this study, almost all of the absolute maximum IM were <1° a the pitch and roll axes for SRS. Therefore, the Encompass can immobilize patients with high accuracy for rotational axes and adequate doses are delivered for HA treatment.

In this study, the HA treatment time with IM_NC_ is shorter than that with corrected IM, because the former method eliminates the process of MV image acquisition and matching with DRR during treatment. However, the absolute maximum shifts in IM were smaller than IM_NC_, and there was no correlation between patient motion and the duration of treatment in this study. Lewis et al. showed a similar relationship between IM and treatment time.^15^ Therefore, it can be said that it is better to correct IM using MV acquisition during HA treatment.

This study had several limitations. First, the number of patients was limited and the performance status (PS) of patients was not considered. If a patient has cognitive deterioration or poor PS, then it will be more difficult for them to stay stationary for a long time. Second, the accuracy of the isocenter, image, and geometry were not considered. Hao et al. evaluated the geometric error to determine whether image registration is a reliable way of monitoring IM at different couch angles and they reported that systematic errors in the image registration varied slightly with the couch angle.^16^ At our institution, the couch motion around the isocenter is checked daily and the geometric error of the couch motion is checked monthly. In addition, in a previous IsoCal the offset between the MV imager center and the treatment isocenter were 0.11 and 0.08 mm for TruebeamSTx and EDGE, respectively, and the kV imager offsets were 0.14 and 0.14 mm, respectively. Third, corrections to the rotational axes were not applied during treatment because MV images were only acquired in the AP and PA directions. In recent years, multi systems have been developed for image guidance, such as 2D/3D image registration in Exactrac (BrainLab AG, Feldkirchen, Germany),^17^ which can correct rotational setup errors based on the 3D volumetric image acquired during treatment. Fourth, the patient may move after the MV image is acquired and there is no confirmation whether the IM occurred before dose delivery. A surface monitor is one method that can be used to control real‐time patient face movements during treatment.^18^ Despite these limitations, our clinical data provide important information that can be used when considering the use of image guidance systems for HA and for careful estimation of the setup error.

## 5 CONCLUSION

This study demonstrated that Encompass provided patient immobilization with adequate accuracy during HA treatment with the absolute maximum displacements for IM <1 mm along the translational axes and <0.5° along the rotational axes. In addition, the average IM was less than IM_NC_ along the translational/rotational axes, and no statistically significant relationship between the treatment time and the IM was observed. Consequently, we believe that MV image acquisition during treatment is useful for HA treatment.

## CONFLICT OF INTEREST

No conflicts of interest.
